# The Mechanical Properties of Gangue Paste Material for Deep Mines: An Experimental and Model Study

**DOI:** 10.3390/ma15175904

**Published:** 2022-08-26

**Authors:** Qiang Leng, Qingliang Chang, Yuantian Sun, Biao Zhang, Jianzhuang Qin

**Affiliations:** 1 School of Mines, China University of Mining and Technology, Xuzhou 221116, China; 2 Key Laboratory of Deep Coal Resource Mining, Ministry of Education, China University of Mining and Technology, Xuzhou 221116, China

**Keywords:** gangue paste material, mechanical properties, response surface

## Abstract

Gangue paste material is mainly composed of coal gangue with particle size, which is mixed with cement. Fly ash and additives can be added to change its performance. In this paper, the influence of each component on the mechanical properties of gangue paste material was studied by an orthogonal experiment. The conversion relationship among various indexes of mechanical properties of gangue paste material and the response surface prediction model were discussed. The results show that the mechanical properties of gangue paste materials are positively correlated with the content of cement, the content of fly ash and the mass concentration, which increase with the increase of the three factors, and show the primary and secondary relationship of the content of cement > the content of fly ash > the mass concentration. A response surface prediction model of mechanical property parameters is established, which includes the first order term of the influencing factors of gangue paste material and the first order interaction term between any two factors. In the response surface prediction model of uniaxial compressive strength, splitting tensile strength, cohesion and elastic modulus, the goodness of fit test coefficients are 0.998, 0.957, 0.970 and 0.997, respectively, which proves that the model has good goodness of fit. The research results provide basic parameters for paste filling mining practice, and also provide the basis for numerical simulation of filling body value.

## 1. Introduction

In the early 21st century, paste filling materials developed rapidly with unique characteristics. The gangue paste has excellent pipeline transportation performance such as high concentration, no critical flow rate, and plunger structure flow. There is no bleeding, segregation, and precipitation in the process of transportation and solidification, and the compression ratio is extremely low after solidification, which accounts for its favourable reception in markets, especially in backfill mining technology [1,2]. The mechanical properties of paste filling materials are closely related to the control effect of surface subsidence deformation. In addition, many scholars have carried out a lot of research on it [3,4,5,6,7,8,9]. The mechanical properties of paste materials are mainly affected by the amount of cement, curing conditions, the ratio of binder to aggregates, and particle size distribution [10,11,12,13,14,15,16]. Through uniaxial compression and triaxial tests, the characteristic information of backfill before loading failure is studied, and four stages of deformation and failure of the stress-strain curve are proposed. The internal relationship of energy dissipation, confining pressure, and stress-strain under different confining pressure loading conditions, as well as crack propagation, macro failure, and fracture surface distribution were analyzed [17,18,19,20,21]. Through response surface analysis, Ensemble Learner Algorithms, or BP neural network, the strength of paste filling material can be obtained by using the mixture ratio of paste filling material [15,18,22,23,24]. Most of the studies only focus on the uniaxial compressive strength and elastic modulus, but few on the tensile strength, cohesion, internal friction angle, and Poisson’s ratio [25,26,27]. Therefore, through the method of orthogonal test, the basic mechanical properties of gangue gypsum body material were comprehensively analyzed. The results are of great significance to the understanding of the strength characteristics of gangue paste filling materials, and also provide a theoretical basis for the selection of filling body parameters in the numerical simulation of gangue gypsum body filling mining.

## 2. Material and Methods

### 2.1. Materials

**Figure 1:**
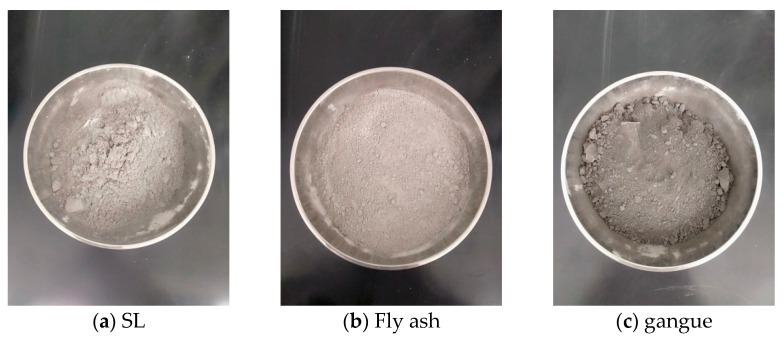
The raw materials.

**Figure 2:**
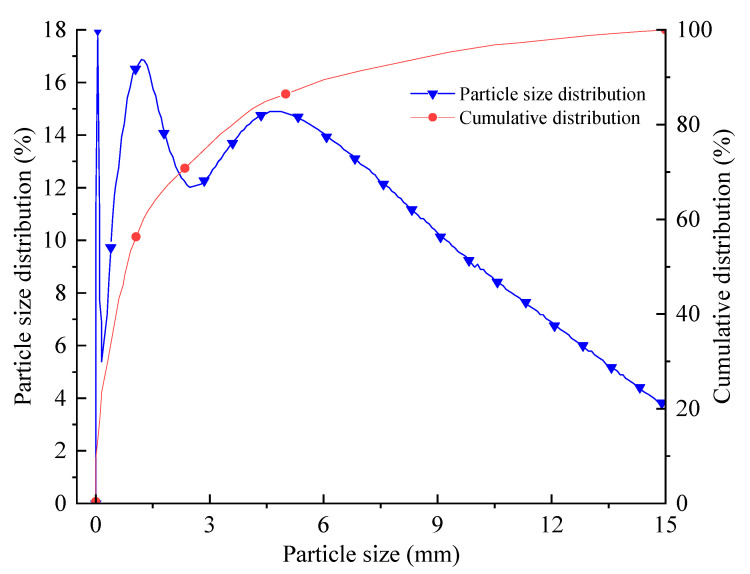
Grain size distribution of gangue in Gaohe coal mine.

The test gangue was taken from the Gaohe coal mine of Shanxi Lu’an Group. Coal gangue is mainly composed of SiO_2_, Al_2_O_3_, and some trace elements. It is a combination of argillaceous, carbonaceous, and sandy shale. Coal gangue is a kind of rock associated with the formation of coal, and its hardness is harder than coal The main components of the fly ash are shown in Table 1; The cement is an SL cement developed by Professor Zhou Huaqiang’s research group of China University of mining and technology (Figure 1). Its physical and mechanical properties are shown in Table 2 and Table 3; The particle size distribution of coal gangue water-screen gradation is shown in Figure 2.The mechanical and working properties of the paste material can be improved by adding the same dosage of bFGF in each group, PA is 3 kg/m^3^, HA is 5 kg/m^3^.

To better study, the influence law of each component of paste material on the mechanical properties of gangue paste material and the conversion relationship and prediction model among parameters, this paper adopts an orthogonal test to set 9 groups of proportion, which can not only consider the interaction of single factor and various factors, but also easily set up multiple groups of the reasonable proportioning scheme. The experimental design was three factors and three levels, and the orthogonal factor levels are shown in Table 4, and the L9 (3^3^) orthogonal experimental table was selected. The orthogonal test scheme is shown in Table 5.

The paste filling material is transported to the downhole through the pipeline, and the low fluidity easily leads to pipe plugging and cannot be pumped. Therefore, good fluidity is very important. Fluidity is usually expressed by slump and expansion in engineering. It is generally considered that when the slump of gangue gypsum body material is ≥240 mm, it has pumpability and can be transported through pipes. The slump and expansion of each proportioned material in Table 5 were tested. In addition, the results show that the slump and expansion of the 9 proportions are 240~268 mm and 410~620 mm, respectively [28]. The flow performance can meet the requirements of engineering practice. The proportion has reference significance for engineering practice.

### 2.2. Experiments

**Figure 3:**
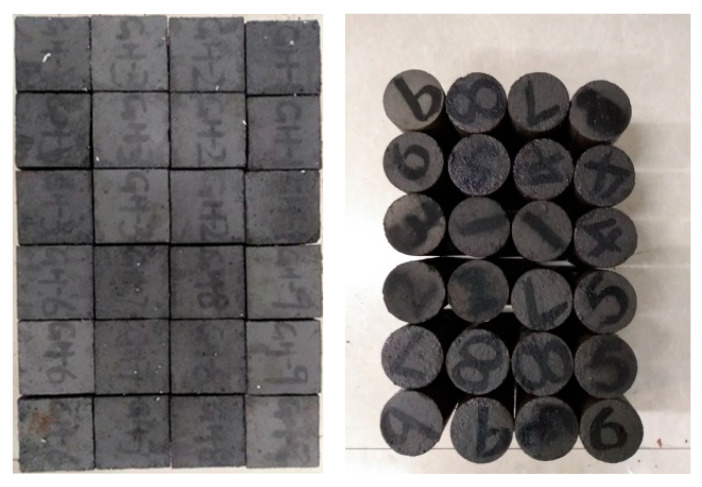
The prepared samples.

In this paper, 81 samples of gangue paste were made. Since there is no standard for mechanical property test of paste materials at present, the sample size and test method shall be determined according to The National Standards of China, The Standard for test method of mechanical properties on ordinary concrete and The Methods for Determining the Physical and Mechanical Properties of Coal and Rock. Among them, there are a total of 54 cubic specimens with a size of 100 mm × 100 mm × 100 mm, and a total of 27 cylindrical specimens with a diameter of 50 mm and a height of 100 mm. After the specimens are made, they are put into the curing box for 28 days, and the corresponding tests are carried out. The prepared samples is given in Figure 3, the purpose of the test piece is shown in Table 6. During the test, the loading speed is controlled as 0.5 mm/min.

## 3. Results and Discussion

### 3.1. Experimental Results

**Figure 4:**
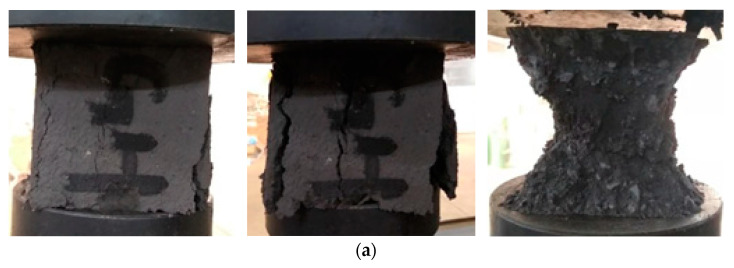

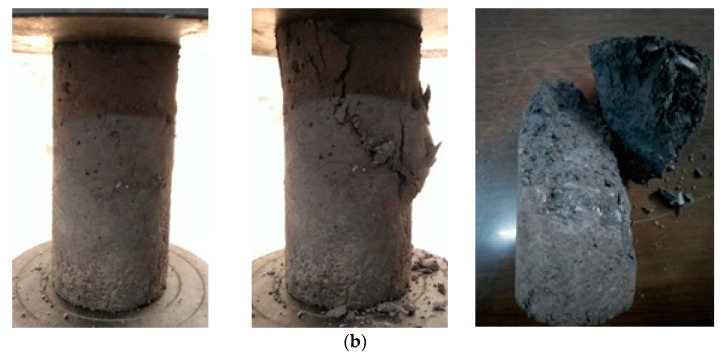
Failure mode of gangue paste material under uniaxial compression. (**a**) Failure mode of cube specimen under compression. (**b**) Failure mode of cylindrical specimen under compression.

The compression failure process of gangue paste material specimen is shown in Figure 4. At the beginning of pressurization, there is no obvious change on the outer surface, and the compression amount in this process is about 2%, and then there are visible cracks. The difference between the cube test piece and the cylinder test piece is the final failure form. After cracks appear in the cube specimen with loading, the four walls protrude outward, and finally show “X” failure characteristics. This is because the cubic gangue gypsum material specimen is affected by the “hoop effect” of the upper and lower pressure plates in the uniaxial compression process. The stress concentration in the middle position is large, and the surface is broken and falls off. It is a typical crushing failure and the compressibility is small. After the cracks appear in the cylindrical specimen with loading, the cracks develop obliquely, and finally penetrate the failure. There are obvious sliding traces on the failure surface, and the failure surface is a small particle cementitious layer, and the large particles are not obviously damaged. This indicates that the cylindrical gangue gypsum body material specimen has a typical shear failure of the cementitious layer during uniaxial compression [29,30].

The arithmetical mean of three specimens for each proportioning scheme is taken as the test value If the difference between the maximum value and the minimum value exceeds 15% of the intermediate value, the maximum value and the minimum value shall be rounded off together, and the intermediate value shall be taken as the compressive strength value of the specimen. If both values exceed 15% of the intermediate value, then the group of tests shall be invalid. The mechanical and deformation performance parameters of the gangue paste are shown in Table 7.

### 3.2. Range Analysis

Range analysis [31,32], also known as intuitive analysis method, is a fast and simple analysis process. It only needs to calculate the average response value $\bar{K_{ij}}$ (j represents factor, i represents level) of different levels of each influencing factor, and calculate the range (R) of corresponding index of each influencing factor level according to the average response value $\bar{K_{ij}}$, and determine the primary and secondary relationship of influencing factors according to the value of R.

It can be seen from Table 8 that in the range analysis of influencing factors of uniaxial compressive strength, splitting tensile strength, cohesion, internal friction angle, and elastic modulus, R _Cement content_ > R _Fly ash content_ > R _Mass concentration_, so the order of influencing factors of the above five indexes of gangue paste material is: cement content, fly ash content, and mass concentration. However, in the Poisson’s ratio range analysis, R _Cement content_ > R _Mass concentration_ > R _Fly ash content_, that is, the primary and secondary factors affecting the Poisson’s ratio of gangue paste material are: cement content, mass concentration and fly ash content.

Uniaxial compressive strength, splitting tensile strength, and elastic modulus increase with the increase of cement content, fly ash content, and mass concentration; The cohesive force increases with the increase of mass concentration and cement content, and increases first and then decreases with the increase of fly ash content, which indicates that fly ash can promote the cohesive force to a certain extent, and the cohesive force of gangue paste material will be reduced if too much fly ash is added; The results show that the influence of cement content, fly ash content, and mass concentration on internal friction angle and Poisson’s ratio is not consistent In the range analysis of this test, the internal friction angle first decreases and then increases with the increase of mass concentration and fly ash content, first increases and then decreases with the increase of cement content, Poisson’s ratio increases first and then decreases with the increase of mass concentration, and decreases with the increase of cement and fly ash.

In the range analysis of the influencing factors of the mechanical properties of gangue paste material, the single factor of cement content, fly ash content, and mass concentration has a positive correlation on most of the mechanical properties, which increases with the increase of the three factors, and shows the primary and secondary relationship of cement content > fly ash content > mass concentration. The influence law of internal friction angle and Poisson’s ratio is not clear in range analysis, which needs further analysis.

### 3.3. Analysis of Variance

The influence of mass concentration [33], cement content and fly ash content on the mechanical properties of gangue paste was discussed by orthogonal test range analysis, and the trend of mechanical properties changing with the level of various factors was analyzed. Since range analysis can not distinguish whether the test results are caused by different levels of factors or by test errors, and can not determine the significance of the influencing factors, and the influence law of internal friction angle and Poisson’s ratio is not clear in range analysis, consequently, variance analysis is carried out for the test results.

According to Table 9, in the variance analysis of factors influencing uniaxial compressive strength, split tensile strength, cohesion, internal friction angle, and elastic modulus, SS _cement material_ > SS _fly ash content_ > SS _mass concentration_, so the main and secondary order of the influencing factors of the above five indexes is: Cement content, fly ash content, and mass concentration, which is consistent with the results of extreme difference analysis. In Poisson ratio extreme difference analysis, SS _cement material_ > SS _mass concentration_ > SS _fly ash content_, that is, the main and secondary order of factors affecting Poisson ratio of gangue gypsum material is: cement content, mass concentration and fly ash content, which is consistent with the results of extreme analysis.

For uniaxial compressive strength, splitting tensile strength, cohesion, and elastic modulus, the F _cement content_ is more than F_0.05_ (2, 8) = 8.65, that is, the change of cement content factor has significant influence on the above four indexes; For internal friction angle and Poisson ratio, the F _mass concentration_ is less than F_0.05_ (2, 8) = 4.46, F _cement content_ is less than F_0.01_ (2, 8) = 8.65, F _fly ash content_ is less than F_0.1_ (2, 8) = 3.11, that is, the change of the three factors of the quality concentration, cement content, and fly ash content has no significant influence on the internal friction angle and Poisson ratio; In internal friction angle, SS _error_ > SS _mass concentration_ indicates that the influence of mass concentration on internal friction angle of material is caused by the error. In Poisson ratio, SS _error_ > SS _cement content_ > SS _mass concentration_ > SS _fly ash content_, which indicates that the influence of mass concentration, cement content, and fly ash content on Poisson ratio of material is also caused by error. Therefore, it can be considered that the influence of cement content, fly ash content, and mass concentration on Poisson’s ratio is positively correlated, and increases with the increase of the three factors, and shows the primary and secondary relationship of cement content > fly ash content > mass concentration.

In the variance analysis of the factors influencing the mechanical properties of gangue paste, it can be seen that the cement content has the most significant influence on the properties; Combined with the analysis of extreme difference, it can be concluded that the factors of cement content, fly ash content, and mass concentration have positive correlation with the mechanical properties of gangue paste, and increases with the increase of the three factors, and shows the primary and secondary relationship of cement content > fly ash content > mass concentration.

### 3.4. Conversion Relationship of Each Index

The quantitative fitting relationship of the compressive strength of the cube and the tensile strength, cohesion, and elastic modulus is statistically analyzed.

(1) Relationship between splitting tensile strength and cube compressive strength.

**Figure 5:**
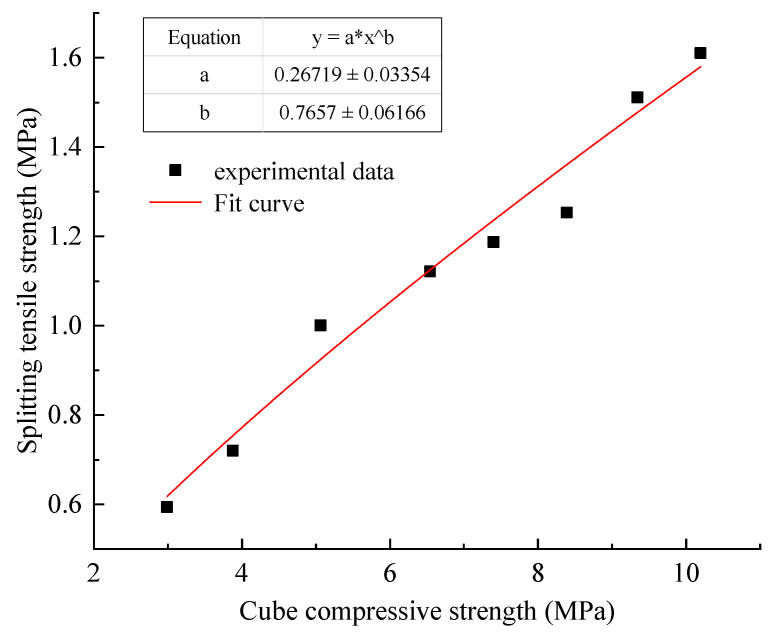
Relationship between cube compressive strength and splitting tensile strength.

As shown in Figure 5, the relationship (1) is obtained by fitting the splitting tensile strength and cube compressive strength of gangue paste material with power function by origin.

(1) $$f_{t}=0.27f_{cu}^{0.77}$$

where $f_{t}$ is Tensile strength (MPa); $f_{cu}$ is Cube Strength (MPa).

Fitting Formula (1) residual sum of squares is 0.030, the goodness of fit R^2^ = 0.966, the fitting effect is better. It can be seen that the uniaxial compressive strength can be used to predict the splitting tensile strength accurately through Formula (1).

(2) Relationship between cohesion and cube compressive strength.

**Figure 6:**
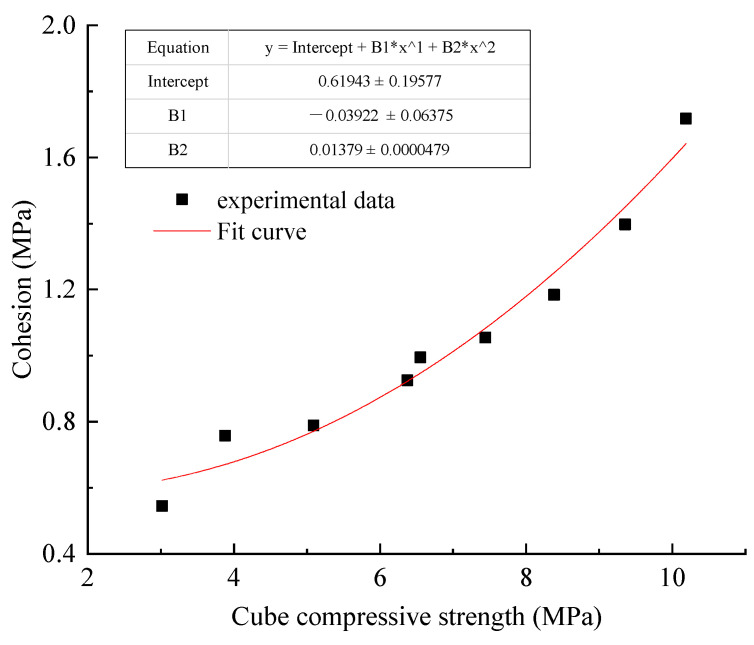
Relationship between cube compressive strength and cohesion.

As shown in Figure 6, the relationship (2) is obtained by fitting the splitting tensile strength and cube compressive strength of gangue paste material with origin.

(2) $$C=0.62-0.04f_{cu}+0.01f_{cu}^{2}$$

where C is cohesion (MPa); $f_{cu}$ is Cube Strength (MPa).

Fitting Formula (2) residual sum of squares is 0.030, goodness of fit R^2^ = 0.971, the fitting effect is better. It can be seen that the cohesive strength value can be accurately predicted by the uniaxial compressive strength value in Formula (2).

(3) Relationship between elastic modulus and cube compressive strength.

**Figure 7:**
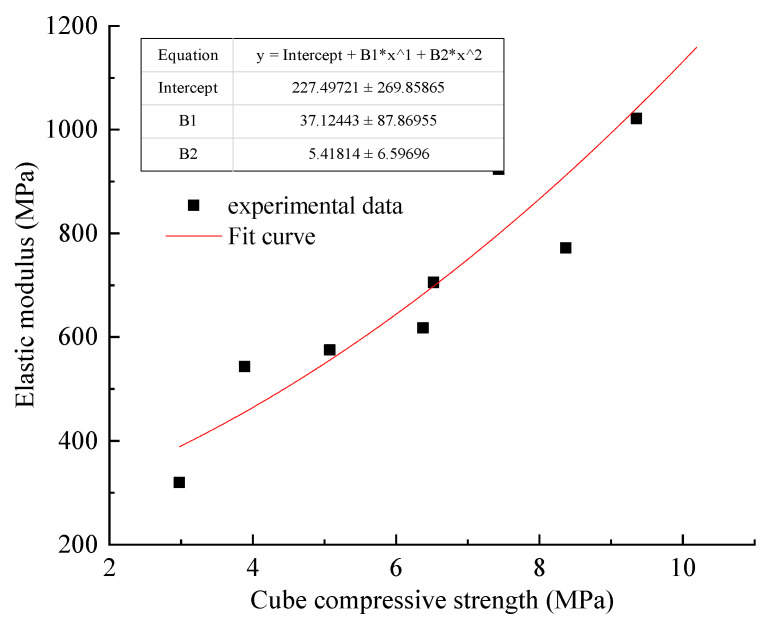
Relationship between cube compressive strength and elastic modulus.

As shown in Figure 7, the relationship (3) is obtained by fitting the elastic modulus and cube compressive strength of gangue gypsum body material with origin.

(3) $$E=227.50+37.12f_{cu}+5.42f_{cu}^{2}$$

where E is Elastic modulus (MPa); $f_{cu}$ is Cube Strength (MPa).

The square sum of residual error of fitting Formula (3) is 25,707.028, and the value is too large. It can be seen that the fitting is not convergent, and the error is large, but the goodness of fit R^2^ = 0.966. Combined with Figure 4, it can be seen that the fitting result has high accuracy, and the value of elastic modulus can be predicted by the uniaxial compressive strength value of Formula (3).

### 3.5. Prediction Model

In this section, based on the data of orthogonal test and response surface analysis by design expert software, a response surface prediction model of mechanical properties parameters is established, which includes the first-order term of the influencing factors of gangue paste material and the first-order interaction term between any two factors. The expressions are shown in (4) to (9).

(4) $$\begin{array}{ll} fc_{u}= & 31.432-41.190x_{1}-0.194x_{2}+0.070x_{3}\\ & +0.560x_{1}x_{2}-0.069x_{1}x_{3}+1.77\times 10^{-5}x_{2}x_{3} \end{array}$$

(5) $$\begin{array}{ll} f_{t}= & -3.320+3.833x_{1}-0.015x_{2}-6.638\times 10^{-3}x_{3}\\ & +0.026x_{1}x_{2}-8.571\times 10^{-3}x_{1}x_{3}+1.143\times 10^{-5}x_{2}x_{3} \end{array}$$

(6) $$\begin{array}{ll} C= & 17.578-21.071x_{1}-0.160x_{2}-0.020x_{3}\\ & +0.197x_{1}x_{2}+0.020x_{1}x_{3}+3.143\times 10^{-5}x_{2}x_{3} \end{array}$$

(7) $$\begin{array}{ll} \varphi = & -1388.313+1686.190x_{1}+9.552x_{2}-7.360x_{3}\\ & -11.371x_{1}x_{2}+8.371x_{1}x_{3}+1.371\times 10^{-3}x_{2}x_{3} \end{array}$$

(8) $$\begin{array}{ll} E= & 24882.275-29820.286x_{1}-217.545x_{2}+104.017x_{3}\\ & +263.920x_{1}x_{2}-121.683x_{1}x_{3}+0.011x_{2}x_{3} \end{array}$$

(9) $$\begin{array}{ll} \mu = & -7.292+8.810x_{1}+0.039x_{2}+0.035x_{3}\\ & -0.046x_{1}x_{2}-0.037x_{1}x_{3}-2.171\times 10^{-5}x_{2}x_{3} \end{array}$$

where *f_cu_* is Uniaxial compressive strength (MPa); *f_t_* is Splitting tensile strength (MPa); *C* is Cohesive force (MPa); *φ* is Internal friction angle (°); *E* is Elastic modulus (MPa); *μ* is Poisson’s ratio; *x*_1_ is Mass concentration; *x*_2_ is Cement content (kg/m^3^); *x*_3_ is Fly ash content (kg/m^3^).

**Figure 8:**
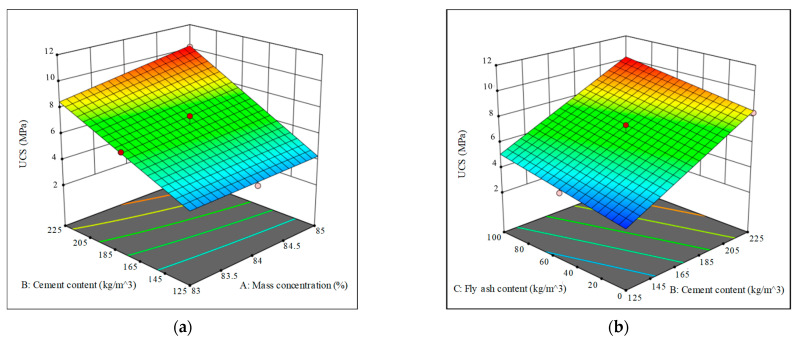

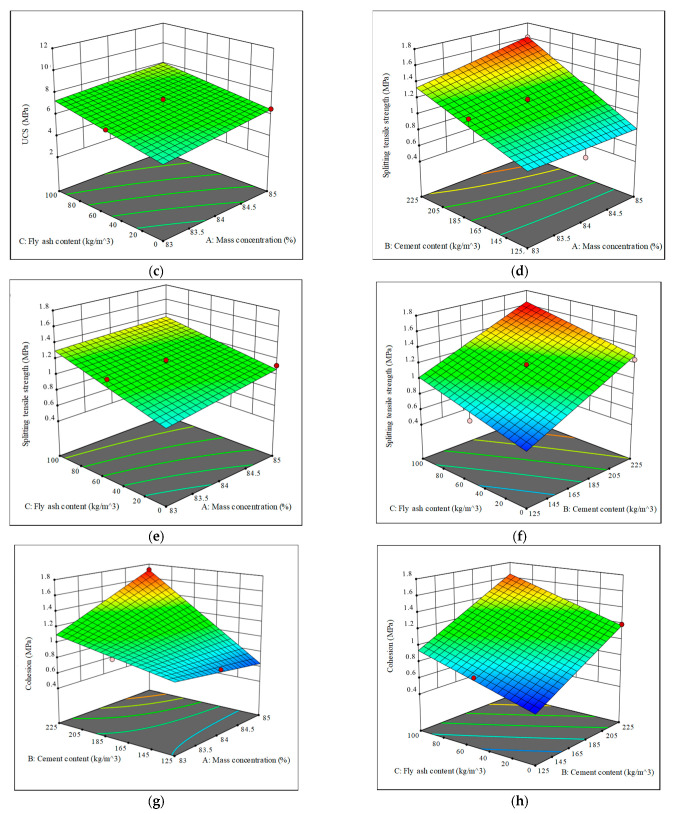

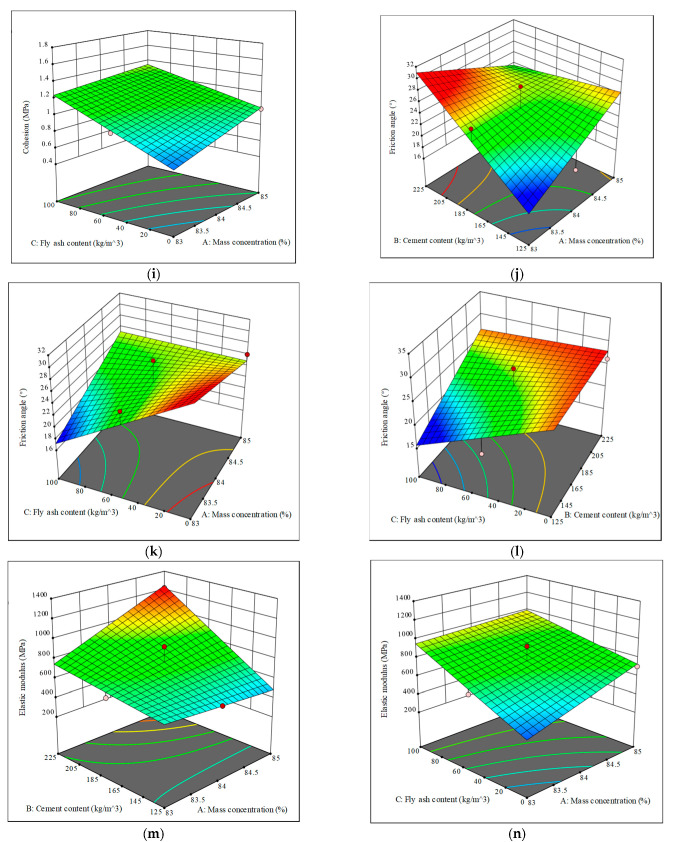

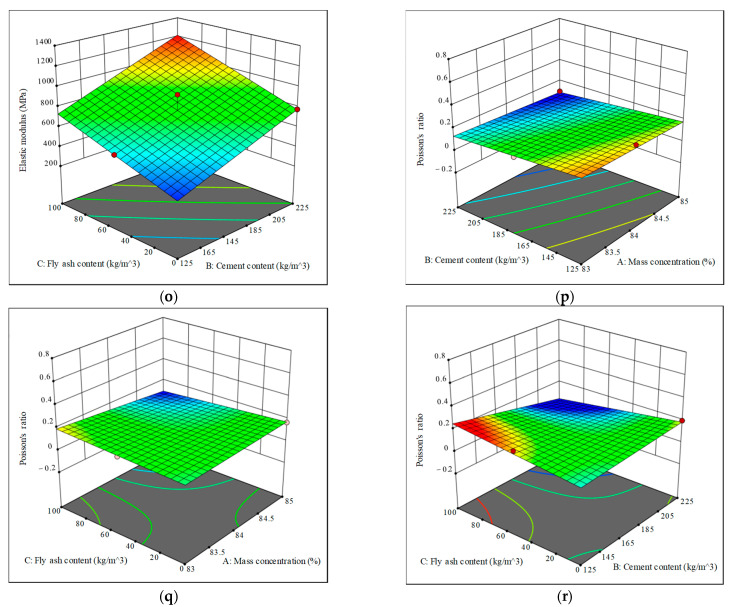
Response surface of different factors to each index.

Design expert software is used to draw the 3D response surface diagram of the three influencing factors of binder content, fly ash content and mass concentration for each response value. The influence results of different factors on uniaxial compressive strength, splitting tensile strength, cohesion, internal friction angle, elastic modulus and Poisson’s ratio are shown in Figure 8. With the help of 3D response surface diagram, we can more intuitively understand the interaction between various influencing factors, and effectively determine the optimal range of different influencing factors.

The results of variance analysis of the response surface prediction model are shown in Table 10 The judgment coefficient R^2^ is the goodness of fit test, which indicates the difference between the measured value and the predicted value. The closer the R^2^ value is to 1, the better the fitting degree is; F is the significance test of the regression equation, which indicates the significance of the fitting equation. The larger the value is, the more significant the equation is; When *p* < 0.05, the factor is significant, when *p* > 0.05, the factor is not significant.

R^2^ = 0.998 in response surface prediction model of uniaxial compressive strength, which proves that the model fits well. P _model_ < 0.05 shows that the whole model is significant, P_X2_ < P_X3_ < 0.05 shows that the amount of cement and fly ash has a significant impact on the prediction model of uniaxial compressive strength, and the cement is the most prominent. The R^2^ = 0.957 in the variance analysis of the response surface prediction model of splitting tensile strength proves that the model has a good fitting degree. R^2^ = 0.970 in variance analysis of cohesive strength response surface prediction model, which proves that the model has a good fitting degree. In the analysis of variance, R^2^ = 0.735, which indicates that the model fitting is low; P _model_ > 0.05, indicating that the overall model is not significant. R^2^ = 0.997 in variance analysis of elastic modulus response surface prediction model, which proves that the model has a good fit; P _model_ < 0.05 shows that the model as a whole is significant, P_X2_ < P_X3_ < P_X1_ < p_x1x2_ < 0.05, indicating that cementitious materials, fly ash content, mass concentration, and the interaction between mass concentration and cementitious materials have significant effects on the prediction model of elastic modulus, and cementitious materials are the most prominent. In the analysis of variance of Poisson’s ratio response surface prediction model, R^2^ = 0.875, which proves that the model fitting is low; P _model_ > 0.05, indicating that the overall model is not significant

## 4. Conclusions

(1) According to the range analysis and variance analysis of the orthogonal experimental results of gangue paste filling material, it can be seen that the influence of single factor of cement content, fly ash content, and mass concentration on each performance index increases with the increase of the three-factor levels, and shows the primary and secondary relationship of cement content > fly ash content > mass concentration.
(2) The uniaxial compressive strength, splitting tensile strength, cohesive force, and elastic modulus of gangue paste material can be fitted by origin, which has a good functional relationship, and this relationship is less affected by mass concentration, fly ash content, and cement content.
(3) Through response surface analysis, the mathematical prediction model of the first-order interaction term between any two factors and the first-order interaction term of the influencing factors of the mechanical and deformation properties of gangue gypsum material is established, and it has high accuracy.
(4) In this paper, the orthogonal experiment is selected in the experiment, and the number of experiments is reduced, which may lead to some shortcomings in the test results. Therefore, more experiments need to be carried out in the future, and the artificial intelligence prediction will be combined to acquire a better model.
(5) The mechanical property prediction model of gangue gypsum body material is used to guide the determination of the proportion parameters of the filling body material in Gaohe coal mine. Through filling mining, a large amount of solid wastes in Gaohe coal mine are treated, and the problems of coal pressing under buildings are also solved.

## Figures and Tables

**Table 1:** Main components and contents of fly ash.

Component	SiO_2_	Al_2_O_3_	CaO	Fe_2_O_3_	C
Content	48.8%	20.3%	2.2%	6.2%	9.9%

**Table 2:** Physical properties of SL cement.

Consistency of Clean Pulp	Volume Stability	Fineness/% 0.08 mm Standard Sieve Residue	Setting Time/h:min	
			Initial Setting	Final Coagulation
23	Stable	4.00	2:00	5:00

**Table 3:** Mechanical properties of SL cement.

Flexural Strength/MPa			UCS/MPa			Flexural Compression Ratio		
3 d	7 d	28 d	3 d	7 d	28 d	3 d	7 d	28 d
6.76	6.85	7.03	37.3	40.9	43.1	0.10	0.17	0.16

**Table 4:** Level table of orthogonal factors.

Level of Orthogonal	Influence Factor		
	Mass Concentration (%)	Cement Content (kg/m^3^)	Fly Ash (kg/m^3^)
Level 1	83	125	0
Level 2	84	175	50
Level 3	85	225	100

**Table 5:** Orthogonal test scheme.

No.	Mass Concentration (%)	Cement Content (kg/m^3^)	Fly Ash (kg/m^3^)	PA (kg/m^3^)	HA (kg/m^3^)	Slump (mm)	Expansion Degree (mm)
GH-1	83	125	0	3	5	240	500
GH-2	83	175	50	3	5	260	580
GH-3	83	225	100	3	5	265	620
GH-4	84	125	50	3	5	255	590
GH-5	84	175	100	3	5	265	550
GH-6	84	225	0	3	5	268	610
GH-7	85	125	100	3	5	255	595
GH-8	85	175	0	3	5	250	510
GH-9	85	225	50	3	5	236	410

**Table 6:** Parameters and application of test piece.

Size	Quantity	Function
100 mm × 100 mm × 100 mm	There are 6 for each ratio, and 54 specimens in total	27 for cube compression test; 27 for splitting tensile test
50 mm × 100 mm cylindrical	There are 3 for each ratio, and 27 specimens in total	Determination of elastic modulus and Poisson’s ratio

**Table 7:** Test values of mechanical parameters of gangue paste materials.

No.	UCS (MPa)	Splitting Tensile Strength (MPa)	Cohesion (MPa)	Friction Angle (°)	Elastic Modulus (MPa)	Poisson’s Ratio
GH-1	3.00	0.60	0.55	25.1	318.20	0.12
GH-2	6.37	1.18	0.93	25.9	618.64	0.16
GH-3	9.33	1.51	1.40	25.9	1028.23	0.10
GH-4	3.89	0.72	0.76	18.0	542.06	0.22
GH-5	7.41	1.19	1.06	28.1	924.58	0.10
GH-6	8.37	1.25	1.19	26.3	778.57	0.20
GH-7	5.07	1.00	0.79	24.3	573.54	0.21
GH-8	6.54	1.12	1.00	26.1	707.08	0.17
GH-9	10.16	1.61	1.72	23.1	1218.87	0.10

**Table 8:** Range analysis of factors influencing mechanical properties of gangue paste materials.

Index	UCS			Splitting Tensile Strength			Cohesion		
	Mass Concentration	Cement Content	Fly Ash Content	Mass Concentration	Cement Content	Fly Ash Content	Mass Concentration	Cement Content	Fly Ash Content
*K*_1j_	6.23	3.99	5.97	1.10	0.77	0.99	0.96	0.70	0.91
*K*_2j_	6.56	6.77	6.81	1.09	1.16	1.17	1.00	1.00	1.14
*K*_3j_	7.26	9.29	7.27	1.24	1.46	1.23	1.17	1.44	1.08
*R*	1.03	5.30	1.30	0.15	0.69	0.24	0.21	0.74	0.23
**Index**	**Friction Angle (°)**			**Elastic Modulus (MPa)**			**Poisson’s Ratio**		
	**Mass Concentration**	**Cement Content**	**Fly Ash Content**	**Mass Concentration**	**Cement Content**	**Fly Ash Content**	**Mass Concentration**	**Cement Content**	**Fly Ash Content**
*K*_1j_	25.6	22.5	25.8	655.02	477.93	601.28	0.13	0.18	0.16
*K*_2j_	24.1	26.7	22.3	748.40	750.10	793.19	0.17	0.14	0.16
*K*_3j_	24.5	25.1	26.1	833.16	1008.56	842.12	0.16	0.13	0.14
*R*	1.5	4.2	3.8	178.14	530.63	240.84	0.04	0.05	0.02

**Table 9:** Variance analysis of factors influencing mechanical properties of gangue paste materials.

Sources of Volatility	UCS (MPa)			Splitting Tensile Strength (MPa)			Cohesion (MPa)		
	Sum of Squares of Deviations (SS)	F Value	Critical Test Value (Fa)	Sum of Squares of Deviations (SS)	F Value	Critical Test Value (Fa)	Sum of Squares of Deviations (SS)	F Value	Critical Test Value (Fa)
Mass concentration	1.642	11.95	F_0.05_ (2, 8) = 4.46	0.059	54.63	F_0.05_ (2, 8) = 4.46	0.074	2.48	F_0.05_ (2, 8) = 4.46
Cement content	42.172	307.03	F_0.01_ (2, 8) = 8.65	0.705	647.53	F_0.01_ (2, 8) = 8.65	0.824	27.70	F_0.01_ (2, 8) = 8.65
Fly ash content	2.605	18.96	F_0.1_ (2, 8) = 3.11	0.096	87.82	F_0.1_ (2, 8) = 3.11	0.082	2.74	F_0.1_ (2, 8) = 3.11
Error, c	0.137			0.011			0.030		
Sum	46.556			0.861			1.010		
Sources of Volatility	Friction Angle (°)			Elastic Modulus (MPa)			Poisson’s Ratio		
	Sum of Squares of Deviations (SS)	F Value	Critical Test Value (Fa)	Sum of Squares of Deviations (SS)	F Value	Critical Test Value (Fa)	Sum of Squares of Deviations (SS)	F Value	Critical Test Value (Fa)
Mass concentration	3.669	0.40	F_0.05_ (2, 8) = 4.46	47,638	1.16	F_0.05_ (2, 8) = 4.46	0.003	0.32	F_0.05_ (2, 8) = 4.46
Cement content	27.416	3.01	F_0.01_ (2, 8) = 8.65	422,436	10.30	F_0.01_ (2, 8) = 8.65	0.004	0.39	F_0.01_ (2, 8) = 8.65
Fly ash content	26.509	2.91	F_0.1_ (2, 8) = 3.11	97,223	2.37	F_0.1_ (2, 8) = 3.11	0.001	0.12	F_0.1_ (2, 8) = 3.11
Error, c	9.109			40,995			0.011		
Sum	66.702			608,292			0.020		

**Table 10:** Variance analysis of prediction model for mechanical properties of gangue paste materials.

Variation Source	UCS (MPa)			Splitting Tensile Strength (MPa)			Cohesion		
	Sum of Squares	*p* Value	R^2^	Sum of Squares	*p* Value	R^2^	Sum of Squares	*p* Value	R^2^
Model	46.450	0.007	0.998	0.820	0.122	0.957	0.980	0.087	0.970
x_1_	0.750	0.062		0.020	0.405		0.055	0.197	
x_2_	18.200	0.003		0.310	0.054		0.370	0.039	
x_3_	1.640	0.030		0.043	0.265		0.047	0.218	
x_1_x_2_	0.091	0.314		1.93 × 10^−4^	0.928		0.011	0.477	
x_1_x_3_	1.371 × 10^−3^	0.885		2.143 × 10^−5^	0.976		1.167 × 10^−4^	0.938	
x_2_x_3_	2.288 × 10^−3^	0.853		9.524 × 10^−4^	0.841		7.202 × 10^−3^	0.560	
Residual	0.100			0.037			0.030		
Net error	46.560			0.860			1.010		
**Variation** **Source**	**Friction Angle (°)**			**Elastic Modulus (MPa)**			**Poisson’s Ratio**		
	**Sum of Squares**	***p* Value**	**R^2^**	**Sum of Squares**	***p* Value**	**R^2^**	**Sum of Squares**	***p* Value**	**R^2^**
Model	65.150	0.604	0.735	6.062 × 10^5^	0.011	0.997	0.017	0.329	0.875
x_1_	3.460	0.642		27,749.060	0.036		2.881 × 10^−4^	0.677	
x_2_	30.520	0.249		1.448 × 10^5^	0.007		3.086 × 10^−3^	0.255	
x_3_	50.270	0.175		91,202.260	0.012		1.61 × 10^−3^	0.372	
x_1_x_2_	37.720	0.215		20,315.680	0.049		6.095 × 10^−4^	0.555	
x_1_x_3_	20.440	0.318		4318.630	0.182		4.024 × 10^−4^	0.626	
x_2_x_3_	13.710	0.393		881.740	0.459		3.438 × 10^−3^	0.237	
Residual	23.550			2127.330			2.467 × 10^−3^		
Net error	88.700			6.083 × 10^5^			0.020		

## Data Availability

Not applicable.
